# Sleep Quality, Not Sleep Duration, Is Independently Associated with Internalized Weight Bias: The Greek Lifestyle and Obesity-Related Bias Survey

**DOI:** 10.3390/clockssleep8030040

**Published:** 2026-06-29

**Authors:** Athina Tzifopoulou, Despoina Dragataki, Maria G. Grammatikopoulou, Eleni C. Pardali, Maria Dimitriou, Dimitrios Poulimeneas

**Affiliations:** 1 Department of Nutritional Science and Dietetics, School of Health Sciences, University of the Peloponnese, Antikalamos, GR-24150 Kalamata, Greece; athina-tzifopoulou@hotmail.com (A.T.); m.dimitriou@go.uop.gr (M.D.); 2 Immunonutrition and Clinical Nutrition Unit, Department of Rheumatology and Clinical Immunology, Faculty of Medicine, School of Health Sciences, University of Thessaly, Biopolis, GR-41223 Larissa, Greece; 3 Department of Nutrition and Dietetics, School of Health Sciences and Education, Harokopio University, Kallithea, GR-17671 Athens, Greece

**Keywords:** weight bias internalization, anti-fat attitudes, weight stigma, insomnia, sleep quality, overweight, self-stigma

## Abstract

Internalized weight bias—the self-directed endorsement of weight-related stereotypes—has emerged as a psychologically potent correlate of health outcomes in individuals with overweight and obesity, yet its relationship with sleep remains largely unexplored. In a cross-sectional manner, 495 Greek adults with a history of overweight/obesity were assessed regarding sleep quality and duration, internalized weight bias (Modified Weight Bias Internalization Scale; WBIS-M), and expressed anti-fat attitudes (Anti-Fat Attitudes Questionnaire, AFA: Dislike, Fear of Fat, Willpower). Insomnia prevalence, assessed with the Athens Insomnia Scale (AIS), was high at 57.6%—nearly doubling across ascending WBIS-M tertiles (39.9% to 73.1%). In hierarchical linear regression models, AIS score remained independently associated with WBIS-M after adjustment for depression, anxiety, BMI, and a comprehensive range of sociodemographic and clinical covariates (B = 0.058; 95% CI: 0.036–0.079; *p* < 0.001), with the fully adjusted model explaining 58.5% of total variance in WBIS-M. AFA subscales did not remain significant in the model post-full adjustment, and sleep duration failed to show independent association with either bias dimensions. The sleep–weight bias association was therefore specific to the internalized dimension and to sleep quality, rather than quantity. These findings highlight a clinically relevant and dimension-specific link between insomnia symptoms and internalized weight stigma, and suggest that routine sleep assessment may be warranted in individuals with a history of overweight or obesity presenting with elevated internalized weight bias—and vice versa.

## 1. Introduction

Adequate sleep is a fundamental component of human health and a key regulator of metabolic homeostasis. A growing body of evidence demonstrates that insufficient sleep duration and poor sleep quality are associated with increased risk of obesity, impaired glucose metabolism, adverse cardiometabolic profiles, and greater all-cause mortality [1,2,3]. Sleep disturbances are highly prevalent among individuals with overweight and obesity, with meta-analytic data indicating increased rates of insomnia symptoms, sleep fragmentation, and reduced sleep efficiency [4,5]. Importantly, sleep is not only a biological process but also a health behavior shaped by psychological, social, and environmental determinants, many of which remain insufficiently explored in populations with a history of overweight or obesity [6].

These individuals are disproportionately exposed to weight bias, a multifaceted construct encompassing negative attitudes, stereotypes, and discriminatory behaviors based on body weight [7]. Emerging evidence suggests that weight bias—particularly its internalized forms—is associated with poorer sleep outcomes. Recent studies have shown that higher levels of internalized weight bias are linked to reduced sleep quality, greater insomnia symptoms, and increased sleep disturbances, with some evidence indicating that these associations may be mediated by psychological factors such as depression and anxiety [8,9]. Similarly, experiences of weight-related stigma have been associated with shorter sleep duration, longer sleep latency, and poorer overall sleep quality, particularly in younger populations, further supporting a potential link between weight bias and sleep health [10]. However, the existing literature remains limited in both the number of research items and scope, with most studies focusing on single dimensions of weight bias and specific population groups. Whether the co-occurrence of expressed and internalized weight bias confers additional burden on sleep outcomes remains unknown.

Weight bias operates across a continuum, ranging from externally expressed negative attitudes (e.g., anti-fat attitudes) to internalized processes whereby individuals endorse and apply weight-related stereotypes to themselves. While both dimensions have been independently associated with adverse psychological and behavioral outcomes—including depressive symptoms, anxiety, and maladaptive health behaviors—their combined role in shaping sleep-related outcomes has not been systematically examined [11,12,13,14]. This represents an important gap, as different dimensions of weight bias may exert distinct or complementary effects on sleep, through shared mechanisms such as psychological distress, heightened cognitive arousal, and stress-related physiological dysregulation. These processes are well-established contributors to sleep disturbances and insomnia [15], and may also interact with circadian regulation pathways affected by chronic stress exposure [16].

Therefore, the aim of the present study was to investigate the association between multiple dimensions of weight bias—including expressed attitudes and internalized forms—and sleep habits in adults with a history of overweight or obesity.

## 2. Results

The descriptive characteristics of the study’s population are presented in Table 1. The sample was predominantly female, with a median age of 38 years. Most participants were highly educated, married or cohabitating with partners, employed in the private sector, and living in large urban areas. Notable sex differences emerged in family and financial status, as well as living arrangements; males were more likely to be single, report income exceeding expenses, and live alone, while females more commonly lived with a spouse or family member and reported greater financial strains. Educational level also differed by sex (*p* = 0.022), driven largely by a greater proportion of doctoral degrees attained among males. Occupation status and place of residence were comparable across sexes.

Table 2 details the history of changes in body weight, the medical history, as well as the measures of weight stigma across the sample. The sample showed largely comparable weight history between sexes, with no significant differences in maximum body mass index (BMI), current BMI, or current-to-maximum weight ratio. Weight stigma measures revealed more pronounced sex differences: females reported significantly greater weight bias internalization (WBIS) as well as AFA Dislike, whereas males scored higher on overall AFA, and specifically on the Fear of Fat and Willpower subscales. Regarding comorbidities, males presented with a higher prevalence of dyslipidemia and excess weight-related conditions, while other cardiometabolic and psychiatric comorbidities were distributed similarly across sexes. Smoking habits did not differ significantly between groups.

Table 3 details characteristics of sleep quality and duration within the sample. Sleep duration was similar across sexes, with the majority of participants reporting adequate sleep and no significant differences in short, adequate, or excess sleep prevalence. Sleep quality, however, differed significantly by sex, with females reporting poorer sleep quality scores on the Athens Insomnia Scale (AIS). Of note, 57.6% of the total sample met the AIS ≥ 6 threshold for insomnia, a notably high prevalence relative to general population estimates, underscoring the relevance of this sample for studying sleep-related outcomes. Despite this, the proportion meeting the threshold for insomnia (AIS ≥ 6) did not reach statistical significance, suggesting that while females tended toward worse sleep quality on a continuous level, the clinical categorization was comparable between groups.

Then, the relationship between sleep habits and measures of weight stigma was explored through hierarchical linear regression models (Table 4). In the crude model, each unit increase in AIS was associated with greater WBIS-M (B = 0.117, *p* < 0.001). This association persisted across progressive covariate adjustment, remaining significant in the fully adjusted model (B = 0.058, 95% CI: 0.036–0.079, *p* < 0.001), which accounted for 58.5% of the variance in WBIS-M. Notably, adjustment for sleep duration did not attenuate the AIS–WBIS association, confirming that sleep quality operates independently of sleep quantity. For the AFA subscales, crude and partially adjusted models yielded significant associations with Fear of Fat and Dislike; however, none survived full adjustment, and the Willpower subscale showed no association at any stage. Sleep quality was thus independently associated with internalized weight stigma, whereas no similar effects on anti-fat attitudes directed toward others were observed.

Having established the independent linear association between sleep quality and WBIS, we sought to determine whether this association translated into clinically meaningful differences in the likelihood of belonging to distinct internalized stigma groups, and whether sleep quantity and insomnia status carried additional independent information beyond sleep quality alone. As presented in Table 5, across sex-dependent WBIS tertiles, a consistent gradient was observed in both sleep- and weight-related measures. Sleep quality deteriorated progressively from lowest to highest tertile, with median AIS scores rising from 4.5 to 8.0 and insomnia prevalence nearly doubling from 39.9% to 73.1%. Sleep duration was also significantly shorter in the highest compared to the lowest tertile, with a higher proportion of adequate sleepers in the lowest group. Both maximum and current BMI increased monotonically across tertiles, as did minimum and desired BMI, indicating that higher internalized stigma was embedded in a broader history of greater weight burden.

To formally test whether sleep characteristics were independently associated with classification into distinct internalized stigma groups, multinomial logistic regression models were fitted with the WBIS tertile as the outcome (Table 6). In the crude model, insomnia was associated with nearly 2.5-fold lower odds of belonging to the highest versus the lowest WBIS tertile (OR = 0.244, 95% CI: 0.153–0.389), with the association persisting after full adjustment (OR = 0.342, 95% CI: 0.179–0.654). Similarly, each unit increase in AIS was associated with significantly greater odds of belonging to a higher WBIS tertile across all comparisons in crude and adjusted models, with the lowest versus highest contrast remaining significant after full adjustment (OR = 1.152, 95% CI: 1.062–1.251). Sleep duration and adequate sleep duration showed no independent association with WBIS tertile membership after adjustment, corroborating the specificity of sleep quality as the operative sleep dimension in relation to internalized weight stigma.

## 3. Discussion

The present study investigated the associations between multiple dimensions of weight bias and sleep outcomes in adults with a history of overweight or obesity. The primary finding was that insomnia symptom severity was independently associated with internalized weight bias after comprehensive covariate adjustment, with a dose–response gradient observed across WBIS-M tertiles in which insomnia prevalence nearly doubled from the lowest to the highest stigma group. Expressed anti-fat attitudes showed no independent associations with any sleep dimension in fully adjusted models, and sleep duration was not associated with either weight bias measure. Collectively, these findings indicate that the relationship between sleep and weight stigma is dimension-specific, with internalized—rather than expressed—forms of weight bias linked to impaired sleep quality, suggesting a mechanism rooted in self-referential psychological processes rather than outwardly directed attitudes.

The 57.6% insomnia prevalence observed here substantially exceeds general population estimates of 10–30% [17] and is consistent with meta-analytic evidence of elevated sleep disturbance in overweight and obesity [18]. Against this backdrop, the independent association between sleep quality and WBIS-M—persisting after adjustment for depression, anxiety, BMI, and sociodemographic factors and explaining a substantial proportion of the variance (58.5%)—is clinically meaningful. Prior studies linking internalized weight stigma to poorer sleep have relied on limited covariate adjustment and, critically, have not accounted for mood disorder diagnoses [8]. The present findings suggest that the sleep–internalized stigma association is not fully explained by comorbid depression or anxiety, and that sleep quality is associated with internalized stigma burden independently of these established psychological correlates.

The mechanisms underlying the differential associations observed across weight bias dimensions warrant explanation. Internalized weight bias involves self-referential processing—the endorsement of weight-related stereotypes as personally applicable—which has been theorized to engage shame, self-criticism, and identity threat [11,12]. These processes are inherently self-referential and are more likely to persist in the absence of external stimuli, making them particularly relevant to the nocturnal period when competing cognitive demands are reduced. These psychological states are posited to sustain nocturnal hyperarousal through mechanisms central to contemporary insomnia models [19,20], and may therefore represent a plausible pathway linking internalized stigma to impaired sleep. Expressed anti-fat attitudes, directed outward at others, do not engage this self-evaluative loop, which may explain the absence of independent associations with sleep in fully adjusted models. The attenuation of crude associations between AIS and AFA Dislike and Fear of Fat after full adjustment likely reflects confounding by shared psychological distress rather than a direct mechanism. The complete absence of association with the Willpower subscale across all models is consistent with this interpretation, as this subscale captures moralistic attributions about others’ volition rather than affective self-appraisal. This pattern is also consistent with evidence indicating that insomnia symptoms are more closely tied to cognitive–emotional processes than sleep duration, which is more strongly shaped by behavioral, occupational, and environmental constraints [21].

These psychological pathways may be reinforced by previously proposed biological mechanisms. Internalized stigma constitutes a chronic psychosocial stressor, and stigma-related stress has been associated with HPA axis dysregulation and altered diurnal cortisol patterns [22]. Disrupted cortisol rhythms have in turn been linked to impaired circadian clock gene entrainment and reduced sleep–wake cycle stability in experimental and observational studies [23,24], suggesting a biological substrate through which self-directed stigma may compound psychological hyperarousal to further disrupt sleep. In this context, internalized weight bias may act as a chronic circadian disruptor through both behavioral (e.g., irregular sleep timing) and biological (e.g., stress-axis dysregulation) pathways, although this remains to be empirically tested. It is important to note that the present study did not collect biological or circadian data, and the abovementioned pathways are proposed as speculative hypotheses to guide future research. Whether internalized weight bias is associated with measurable circadian misalignment remains untested; objective circadian phenotyping in weight-stigmatized populations represents a direct extension of the present findings.

Sex differences in this sample were consistent with the literature: females reported higher internalized weight bias, in line with evidence of greater sociocultural pressure on women regarding body weight and appearance [7]. The absence of a significant difference in insomnia prevalence at the clinical threshold despite higher continuous AIS scores in females suggests that sex differences in sleep disturbance may be more pronounced at the level of symptom severity than at the level of clinical categorization. The use of sex-specific WBIS-M tertiles was therefore appropriate to ensure valid comparison of stigma–sleep associations across equivalent positions within the internalized bias distribution.

Strengths of this study include the simultaneous assessment of internalized and expressed weight bias dimensions, the use of validated instruments across all constructs, and the application of hierarchical regression models with unusually comprehensive covariate adjustment; the use of sex-stratified tertiles further enhanced interpretability and accounted for known distributional differences in internalized weight bias between sexes. The main limitation is the cross-sectional design, which precludes causal inference regarding the directionality of the sleep–stigma association. Additionally, sleep duration and sleep quality were assessed over different reference periods—the previous week and the previous month, respectively—which may have introduced temporal incongruence between the two measures and should be considered when interpreting findings that involve both dimensions simultaneously. The low prevalence of extreme sleep duration categories—particularly excessive sleep (1.4%)—limited statistical power to detect associations at the tails of the distribution and precluded formal testing of potential non-linear or U-shaped relationships between sleep duration and weight bias measures. The predominantly female, educated, and urban sample composition limits generalizability. Furthermore, the exclusion of pregnant women and those with a recent pregnancy means that the findings cannot be generalized to perinatal populations. Depression and anxiety were captured as self-reported diagnoses rather than validated symptom scales, a further potential source of residual confounding. Similarly, physical comorbidities were recorded as binary variables reflecting diagnosis rather than severity or duration, which may result in residual confounding by the degree of metabolic burden. The present study did not assess habitual screen time, which represents a potentially important behavioral covariate given its established associations with both sleep quality and metabolic outcomes, and its absence may represent a source of residual confounding. Finally, all measures relied on self-reporting—sleep quality and duration, anthropometric history, and weight bias itself—without objective validation, which may have introduced shared method variance and recall bias and could have inflated observed associations; recalled maximum and minimum body weight are particularly susceptible to recall bias, and desired BMI may additionally reflect weight-related self-appraisal processes that overlap conceptually with the internalized weight bias construct itself. Future studies incorporating objective sleep assessment methods, such as actigraphy or polysomnography, would strengthen the validity of these findings and allow for direct comparison with subjectively reported sleep outcomes.

Clinically, these findings suggest that assessment of internalized weight bias may be relevant in individuals presenting with sleep complaints in the context of overweight or obesity. Conversely, sleep-focused interventions—including cognitive behavioral therapy for insomnia, the recommended first-line treatment for chronic insomnia [25]—may warrant consideration in individuals with high internalized weight bias, and vice versa. Whether improvement in sleep quality would attenuate internalized stigma or whether reducing stigma burden would improve sleep cannot be determined from the present cross-sectional data and require longitudinal and interventional investigation. Future longitudinal studies employing validated continuous measures of psychological symptom severity would be well-positioned to formally test mediation pathways linking internalized weight bias, sleep disturbance, and psychological comorbidity. Taken together, the findings support a conceptual model in which internalized weight bias and sleep disturbance may form a mutually reinforcing cycle, with implications for both psychological well-being and long-term weight-related health outcomes.

## 4. Materials and Methods

### 4.1. Study Description

The Greek Lifestyle and Obesity-Related Bias Survey is a cross-sectional study, with the primary aim of exploring the relationship between lifestyle indices and weight bias measures among Greek adults 18–65 years old with a history of excess body weight. The survey was delivered online, with data collected and managed using REDCap (REDCap 14.0.19) [26,27]. Participation in the study was voluntary; all adults were aged 18–65 years old, with a maximum adult BMI ≥ 25 kg/m^2^; all who provided consent were eligible to enroll; and pregnant women (currently or within the previous year) were excluded from sampling. A description of the study has been previously provided [28]. Participants were recruited via a convenience sampling approach, through promotion of the study on social media platforms, patient advocacy groups, dietitian networks, and the University of the Peloponnese newsletter and mailing list. Recruitment was performed in waves; data for the present analysis were drawn from the first two recruitment waves (first wave: May–July 2024; second wave: November 2024–April 2025). Prior to analysis, all submissions were screened for duplicate entries based on response consistency across sociodemographic and anthropometric fields; no duplicates were identified.

### 4.2. Questionnaires and Measures

#### 4.2.1. Sociodemographics

Participants were asked to report their age (in years), sex (male/female), educational status (primary or secondary education: Bachelor’s/Master’s/Doctorate), family status (single/in a relationship—non-married/married or cohabitating/married—separated/divorced/widowed), employment status (public sector/private sector/freelancer/student/pensioner/work without pay (i.e., in family business)/unemployed), economic status (5-point Likert-scale assessing the relationship between income and expenses, ranging from income being much lower than expenses to income being much higher than expenses), and area of residence (large city/smaller city or town/semi-rural area/rural area).

#### 4.2.2. Body Weight History and Smoking Status

Body weight history included current anthropometry (weight, height) and minimum, maximum and desired weight in adult life. Furthermore, participants were asked if they had tried to reduce their weight in the previous year, as well as their weight loss method (diet, physical activity, drug therapy, metabolic surgery or any combination of the above), and how they achieved weight loss (with the assistance of a registered dietician/Medical Doctor/other professional, with assistance from friends and/or family, or by myself). Smoking status was assessed with binary questions regarding current smoking and/or vaping, and smoking cessation.

#### 4.2.3. Sleep Quantity and Quality

Sleep duration was recorded as an average of sleep duration (hours/day) during the previous week. Sleep duration was then classified as short (<6 h per day), adequate (7–9 h per day), or excess (≥10 h per day) following the relative guidance by the National Sleep Foundation [29]. For regression analyses, a binary adequate sleep variable was derived contrasting the recommended 7–9 h category against all other duration groups, including those in the possibly adequate range.

Sleep quality was assessed via the validated Athens Insomnia Scale (AIS). AIS consists of a composite index assessing 8 sleep domains (5 on nocturnal sleep, 3 on daytime dysfunction) that evaluates the severity of perceived insomnia symptoms during the previous month. Participants were asked to rate the frequency of symptoms on a scale ranging from 0 (no reported problem) to 3 (intense reported problems). A composite score is tabulated by summing individual ratings. Thus, the AIS score ranges from 0 to 24, with higher scores indicating worse sleep quality, with scores ≥ 6 indicating the presence of insomnia [30].

#### 4.2.4. Weight Bias Measures

The Greek validated version of the Modified Weight Bias Internalization Scale (WBIS-M) was used to assess the level of internalization of stereotypes related to one’s weight [11,31]. WBIS-M has 11 items, rated on a 7-point Likert scale (1, strongly disagree, to 7, strongly agree). The total WBIS-M score occurs after dividing the sum of each item rating by 11, with the total score ranging from 1 to 7 (higher scores indicating higher internalization of weight-related bias).

Anti-fat attitudes were assessed with the Greek validated Anti-Fat Attitudes Questionnaire (AFA). AFA consists of a total of 13 items, divided into 3 subscales, namely Dislike (7 items), Fear of Fat (3 items) and Willpower (3 items). Each item assessed is rated on a Likert scale ranging from 1 (very strongly disagree) to 9 (very strongly agree). A composite score for each subscale is then tabulated by dividing the sum of each subscale by the N of items of each subscale. The subscale score ranges from 1 to 9, with higher scores indicative of higher domain-specific prejudice against persons with excess body weight [32].

### 4.3. Statistical Analyses

Normality of data was explored graphically with Q-Q plots. According to data distribution, continuous variables were presented as means ± standard deviations (SDs) for normally distributed variables, or else as medians with their respective quartiles (Q1, Q3). All analyses were conducted on a complete case basis. Of the 613 participants who accessed the survey, 495 (80.8%) had complete data on all variables, and thus constituted the analytic sample. Participants with complete versus incomplete data did not differ significantly in age, sex, maximum or current BMI, demographics, weight and medical history (all *p* > 0.05), suggesting that missing data were unlikely to introduce systematic bias. Differences between continuous variables were examined with the independent *t*-test, or Mann–Whitney U test, based on data distribution. Where overall group differences in continuous variables were significant, pairwise comparisons between tertiles were performed using Mann–Whitney U tests with Bonferroni correction. For categorical variables, overall between-group differences were assessed using chi-square tests; pairwise comparisons were not performed.

To examine the association between sleep quality and weight bias measures, hierarchical linear regression models were constructed with each weight bias measure (WBIS-M; AFA: Fear of Fat, Dislike, Willpower) as the dependent variable and AIS score as the primary predictor. Three sequential models were fitted: (i) a crude model; (ii) a model adjusted for sex, age, current BMI, and sleep duration; and (iii) a fully adjusted model additionally incorporating sociodemographic (education, income, relationship status, living arrangements, place of residence), clinical (smoking status, obesity-related comorbidities, depression, anxiety diagnosis), and weight-related (maximum BMI, AFA subscales or WBIS-M, as appropriate) covariates. Covariate selection was guided by theoretical relevance and the prior literature. Variance inflation factors (VIFs) were examined in all fully adjusted models and were all below 5, indicating no meaningful multicollinearity among covariates. To determine whether sleep characteristics were independently associated with classification into distinct internalized stigma groups, the sample was stratified into sex-dependent WBIS-M tertiles. Multinomial logistic regression models were then fitted with tertile membership as the outcome (lowest tertile as reference), with AIS score, insomnia status (AIS ≥ 6), sleep duration, and adequate sleep duration entered separately as predictors across crude, adjusted, and fully adjusted models, using the same covariate structure as above. Significance was set at a = 0.05.

## Figures and Tables

**Table 1:** Descriptive characteristics of the study sample, by sex (N = 495).

		Total Sample (N = 495)	Females (*n* = 349)	Males (*n* = 146)	*p*
Age (years)		38.0 (23.0, 51.0)	39.0 (23.0, 51.0)	34.0 (23.0, 51.0)	0.413
Educational Level (%)	Primary Education	1.2	0.9	2.1	0.022
	Secondary Education	16.4	15.5	18.5	
	Vocational Studies	20.4	20.6	19.9	
	Bachelor’s Degree	41.2	42.4	38.4	
	Postgraduate Degree	18.2	19.5	15.1	
	Doctorate Degree	2.6	1.1	6.2	
Family Status (%)	Single	30.1	24.1	44.5	<0.001
	In a relationship	24.8	26.9	19.9	
	Married/Cohabitating	38.6	41.5	31.5	
	Divorced	5.1	6.0	2.7	
	Widowed	1.4	1.4	1.4	
Employment status (%)	Public Sector	20.0	19.8	20.5	0.591
	Private Sector	33.5	33.5	33.6	
	Freelancer	13.5	12.0	17.1	
	Student	21.0	22.9	16.4	
	Unemployed	5.1	5.2	4.8	
	Pensioner	3.8	3.4	4.8	
	Other	3.0	3.2	2.7	
Financial Status (%)	Income << Expenses	12.7	15.2	6.8	<0.001
	Income < Expenses	11.5	12.9	8.2	
	Income ≈ Expenses	39.4	42.1	32.9	
	Income > Expenses	29.1	24.4	40.4	
	Income >> Expenses	7.3	5.4	11.6	
Living Arrangements (%)	Living alone	22.6	18.9	31.5	0.006
	Living with spouse/family	73.9	77.7	65.1	
	Living with friends/roommates	3.4	3.4	3.4	
Place of Residence (%)	Urban, large city	77.2	76.8	78.1	0.591
	Urban, town	13.7	14.3	12.3	
	Rural	9.1	8.9	9.6	

**Table 2:** Weight history, weight bias and medical history of the sample, by sex (N = 495).

	Total Sample (N = 495)	Females (*n* = 349)	Males (*n* = 146)	*p*
Max BMI (kg/m^2^)	30.0 (27.0, 34.4)	29.7 (26.9, 34.5)	30.6 (27.4, 34.4)	0.582
Max BMI: Overweight (%)	50.2	51.1	48.9	0.516
Max BMI: Obesity (%)	49.8	47.9	52.1	
Current BMI (kg/m^2^)	27.8 (25.2, 32.0)	27.7 (25.1, 32.3)	28.2 (25.6, 31.6)	0.536
Current BMI: Underweight (%)	0.4	0.3	0.7	0.465
Current BMI: Normoweight (%)	22.0	23.7	17.8	
Current BMI: Overweight (%)	43.3	41.9	46.6	
Current BMI: Obesity (%)	34.3	34.1	34.9	
Current-to-Max Weight Ratio (%)	95.9 (88.5, 100.0)	95.8 (88.6, 100.0)	96.1 (88.3, 100.0)	0.979
Weight Stigma Measures				
WBIS–M (1–7)	3.09 (2.18, 4.27)	**3.45 (2.36, 4.59)**	**2.55 (1.73, 3.39)**	<0.001
AFA: Total (1–9)	3.54 (2.62, 4.54)	**3.46 (2.62, 4.38)**	**3.77 (2.77, 4.85)**	0.034
AFA: Fear of Fat (1–9)	2.00 (1.29, 3.00)	**1.86 (1.14, 2.71)**	**2.29 (1.57, 3.71)**	<0.001
AFA: Dislike (1–9)	5.00 (3.00, 7.00)	**5.33 (3.67, 7.00)**	**4.67 (2.58, 6.67)**	0.005
AFA: Willpower (1–9)	5.33 (3.33, 6.67)	**5.00 (3.33, 6.33)**	**6.33 (3.92, 7.33)**	<0.001
Comorbidities (%)				
T2DM	5.1	4.9	5.5	0.778
Hypertension	9.1	7.7	12.3	0.105
CVD (any)	1.8	1.7	2.1	0.799
Dyslipidemia	9.5	**7.7**	**13.7**	0.039
Anything Excess Weight-Related	18.0	**15.8**	**23.3**	0.047
Depression	7.3	8.0	5.5	0.320
Anxiety Disorder	10.5	10.0	11.6	0.593
Smoking Habits (%)				
Actively Smoking and/or Vaping	36.4	36.4	36.3	0.985
In Cessation	11.7	10.3	15.1	0.134
Never Smoked/Vaped	52.1	53.3	49.3	0.419

Bold font denotes significant difference between sexes; AFA, Anti-Fat Attitudes Questionnaire; BMI, body mass index; CVD, cardiovascular disease; T2DM, Type 2 Diabetes Mellitus; WBIS-M, Weight Bias Internalization Scale—Modified.

**Table 3:** Sleep duration and quality of the sample, by sex (N = 495).

	Total Sample (N = 495)	Females (*n* = 349)	Males (*n* = 146)	*p*
Sleep Duration (h/day)	7.0 (6.0, 7.0)	7.0 (6.0, 7.0)	6.5 (6.0, 7.0)	0.763
Short Sleep Duration ^1^ (%)	17.6	17.8	17.1	0.864
Adequate Sleep Duration ^2^ (%)	48.7	49.9	45.9	0.421
Excess Sleep Duration ^3^ (5)	1.4	1.1	2.1	0.435
Sleep Quality (AIS, 0–24)	6.0 (3.0, 9.0)	**7.0 (4.0, 10.0)**	**6.0 (3.0, 9.0)**	0.010
Insomnia ^4^ (%)	57.6	59.3	53.4	0.227

Bold font denotes significant difference between sexes; AIS, Athens Insomnia Scale; ^1^ <6 h per day; ^2^ 7–9 h/day; ^3^ ≥10 h per day; ^4^ AIS ≥ 6.

**Table 4:** Linear regression models exploring correlates of weight stigma measures (N = 495).

**Dependent variable** **WBIS-M (1–7)**	**Model**	**Adjusted R^2^**	**B**	**95% CI**	***p***
AIS (per 1 unit)	Crude	0.133	**0.117**	**0.091, 0.144**	**<0.001**
	Adjusted ^1^	0.329	**0.085**	**0.059, 0.111**	**<0.001**
	Fully adjusted ^2^	0.585	**0.058**	**0.036, 0.079**	**<0.001**
**Dependent variable** **AFA: Fear of Fat (1–9)**	**Model**	**Adjusted R^2^**	**B**	**95% CI**	***p***
AIS (per 1 unit)	Crude	0.014	**0.036**	**0.009, 0.063**	**0.009**
	Adjusted ^1^	0.060	**0.045**	**0.015, 0.074**	**0.003**
	Fully adjusted ^3^	0.302	0.019	−0.009, 0.046	0.180
**Dependent variable** **AFA: Dislike (1–9)**	**Model**	**Adjusted R^2^**	**B**	**95% CI**	***p***
AIS (per 1 unit)	Crude	0.039	**0.111**	**0.063, 0.159**	**<0.001**
	Adjusted ^1^	0.067	**0.081**	**0.029, 0.134**	**0.002**
	Fully adjusted ^4^	0.484	−0.026	−0.067, 0.016	0.223
**Dependent variable** **AFA: Willpower (1–9)**	**Model**	**Adjusted R^2^**	**B**	**95% CI**	***p***
AIS (per 1 unit)	Crude	0.001	0.018	−0.027, 0.062	0.441
	Adjusted ^1^	0.071	0.021	−0.027, 0.069	0.382
	Fully adjusted ^5^	0.313	−0.010	−0.054, 0.034	0.647

Bold font denotes significant associations; AFA, Anti-Fat Attitudes Questionnaire; AIS, Athens Insomnia Scale; BMI, body mass index; CI, confidence interval; WBIS-M, Weight Bias Internalization Scale—Modified; ^1^ adjusted for sex (male vs. female), age (per year), current BMI (per kg/m^2^), and sleep duration (per hour); ^2^ further adjusted for current smoking and/or vaping (vs. not), having any obesity-related comorbidity (vs. not), having tertiary education (vs. not), having greater income than expenses (vs. not), being married or in a relationship (vs. not), living alone (vs. not), living in urban residence (vs. not), reported depression diagnosis (vs. not), reported anxiety disorder diagnosis (vs. not), maximum BMI (per kg/m^2^), and AFA subscales (per 1 unit); ^3,4,5^ similar to adjusted model, with the addition of WBIS-M in all models and removal of the relative AFA subscale that is explored as a dependent variable.

**Table 5:** Demographics, weight-related measures and sleep habits, by sex-dependent WBIS-M tertiles (N = 495).

	Sex-Dependent WBIS-M Tertiles						
	Lowest (*n* = 168)	Medium (*n* = 167)	Highest (*n* = 160)	*p*	*p*_L*Vs*M_	*p*_M*Vs*H_	*p*_L*Vs*H_
Sex (% women)	71.4	70.7	69.4	0.919			
Age (years)	**43.5 (26.0, 54.5)**	**35.0 (22.0, 51.0)**	**34.5 (23.0, 48.0)**	0.002	0.011	0.999	0.004
Married/In Relationship (%)	**67.9**	**68.3**	**53.8**	0.008			
Higher Education (%)	53.8	67.1	60.6	0.234			
Higher Income (%)	**42.3**	**37.7**	**28.7**	0.036			
Living Alone (%)	25.6	27.7	23.8	0.281			
Max BMI (kg/m^2^)	27.8 (26.1, 31.1)	29.8 (27.0, 33.5)	33.2 (29.1, 40.0)	<0.001	**0.001**	**<0.001**	**<0.001**
Current BMI (kg/m^2^)	26.1 (24.1, 28.6)	27.8 (25.2, 31.3)	31.0 (27.4, 37.7)	<0.001	**0.001**	**<0.001**	**<0.001**
Current: Max Weight (%)	95.5 (86.3, 100)	96.6 (88.2, 100)	96.3 (90.6, 100)	0.396	-	-	-
Min BMI (kg/m^2^)	**22.5 (20.7, 24.2)**	23.1 (21.5, 24.7)	**23.5 (21.6, 26.4)**	0.001	0.251	0.175	0.001
Desired BMI (kg/m^2^)	24.1 (22.7, 26.0)	24.3 (22.6, 26.0)	24.8 (23.0, 26.9)	0.035	0.999	0.200	**0.037**
Sleep Duration (h/day)	**7.0 (6.0, 7.4)**	6.0 (6.0, 7.0)	**6.0 (6.0, 7.0)**	0.017	0.068	0.999	**0.027**
Short (%)	13.7	20.4	18.8	0.247	-	-	-
Adequate (%)	**56.5**	**46.1**	**43.1**	**0.037**			
Excess (%)	2.4	1.2	0.6	0.387			
Sleep Quality (0–24)	**4.5 (2.0, 7.0)**	**6.0 (4.0, 9.0)**	**8.0 (5.0, 11.0)**	<0.001	**<0.001**	**0.011**	**<0.001**
Insomnia (%)	39.9	60.5	73.1	<0.001			
WBIS–M (1–7)	**1.81 (1.55, 2.25)**	**3.18 (2.73, 3.73)**	**4.82 (4.37, 5.36)**	<0.001	**<0.001**	**<0.001**	**<0.001**
AFA: Fear of Fat (1–9)	**1.57 (1.00, 2.29)**	**2.00 (1.29, 3.00)**	**2.57 (1.57, 3.86)**	<0.001	**0.007**	**0.006**	**<0.001**
AFA: Dislike (1–9)	**3.33 (1.67, 5.33)**	**5.33 (4.00, 6.67)**	**7.00 (5.33, 8.33)**	<0.001	**<0.001**	**<0.001**	**<0.001**
AFA: Willpower (1–9)	**4.33 (2.67, 6.33)**	**5.33 (4.00, 6.67)**	**5.67 (3.67, 7.00)**	0.002	**0.008**	0.999	**0.007**

Bold font denotes significant associations; AFA, Anti-Fat Attitudes Questionnaire; BMI, body mass index; LVsH, low versus high; LVsM, low versus medium; MVsH, medium versus high; WBIS-M, Weight Bias Internalization Scale—Modified.

**Table 6:** Multinomial logistic regression models exploring associations between sleep habits and classification in sex-dependent WBIS-M tertiles (N = 495).

		Sex-Dependent WBIS-M Tertiles					
		Lowest (Reference)		Medium vs. Lowest		Highest vs. Lowest	
Variable	Model	OR	95% CI	OR	95% CI	OR	95% CI
AIS (per 1 unit)	Crude	1.00	reference	**1.133**	**1.069, 1.201**	**1.208**	**1.139, 1.282**
	Adjusted ^1^	1.00	reference	**1.104**	**1.036, 1.176**	**1.176**	**1.100, 1.258**
	Fully adjusted ^2^	1.00	reference	**1.103**	**1.029, 1.183**	**1.152**	**1.062, 1.251**
Insomnia (% no vs. yes)	Crude	1.00	reference	**0.433**	**0.280, 0.672**	**0.244**	**0.153, 0.389**
	Adjusted ^1^	1.00	reference	**0.491**	**0.305, 0.791**	**0.285**	**0.165, 0.490**
	Fully adjusted ^2^	1.00	reference	**0.552**	**0.328, 0.930**	**0.342**	**0.179, 0.654**
Sleep Duration (per h)	Crude	1.00	reference	1.017	0.846, 1.221	**1.267**	**1.055, 1.523**
	Adjusted ^3^	1.00	reference	0.898	0.730, 1.106	0.988	0.799, 1.248
	Fully adjusted ^4^	1.00	reference	0.919	0.727, 1.161	1.047	0.798, 1.374
Adequate Sleep (% no vs. yes)	Crude	1.00	reference	1.521	0.989, 2.340	**1.716**	**1.109, 2.657**
	Adjusted ^3^	1.00	reference	1.056	0.645, 1.731	0.966	0.559, 1.669
	Fully adjusted ^4^	1.00	reference	1.040	0.607, 1.782	0.942	0.489, 1.817

Bold font denotes significant associations. AFA, Anti-Fat Attitudes Questionnaire; AIS, Athens Insomnia Scale; BMI, body mass index; CI, confidence interval; OR, odds ratio; WBIS-M, Weight Bias Internalization Scale—Modified; ^1^ adjusted for age (per year), current body mass index (per kg/m^2^), and sleep duration (per hour); ^2^ adjusted for ^1^ and further adjusted for current smoking and/or vaping (vs. not), having any obesity-related comorbidity (vs. not), having tertiary education (vs. not), having greater income than expenses (vs. not), being married or in a relationship (vs. not), living alone (vs. not), living in urban residence (vs. not), reported depression diagnosis (vs. not), reported anxiety disorder diagnosis (vs. not), maximum BMI (per kg/m^2^), and AFA subscales (per 1 unit); ^3^ adjusted for age (per year), current BMI (per kg/m^2^), and sleep quality (AIS, per unit); ^4^ adjusted for ^3^ and further adjusted for current smoking and/or vaping (vs. not), having any obesity-related comorbidity (vs. not), having tertiary education (vs. not), having greater income than expenses (vs. not), being married or in a relationship (vs. not), living alone (vs. not), living in urban residence (vs. not), reported depression diagnosis (vs. not), reported anxiety disorder diagnosis (vs. not), maximum BMI (per kg/m^2^), and AFA subscales (per 1 unit).

## Data Availability

Data may be available upon reasonable request submitted to the corresponding author (D.P.), due to restrictions related to legal or ethical reasons.

## Abbreviations

The following abbreviations are used in this manuscript:

AFA	Anti-Fat Attitudes
AIS	Athens Insomnia Scale
BMI	Body mass index
CI	Confidence interval
CVD	Cardiovascular disease
OR	Odds ratio
T2DM	Type 2 Diabetes Mellitus
WBIS-M	Weight Bias Internalization Scale—Modified
